# Rapid emergence from dexmedetomidine sedation in Sprague Dawley rats by repurposing an α_2_-adrenergic receptor competitive antagonist in combination with caffeine

**DOI:** 10.1186/s12871-023-01986-5

**Published:** 2023-02-01

**Authors:** Zheng Xie, Aaron P. Fox

**Affiliations:** 1 Department of Anesthesia and Critical Care, The University of Chicago, Chicago, IL USA; 2 Department of Neurobiology, Pharmacology and Physiology, The University of Chicago, Chicago, IL USA

**Keywords:** Dexmedetomidine, Atipamezole, Sedation, Emergence from sedation, cAMP, α_2_ receptor agonist, α_2_ receptor antagonist, Caffeine, Forskolin

## Abstract

**Background:**

The α_2_ adrenergic receptor agonist dexmedetomidine is an important intravenous sedative with analgesic properties. Currently available dexmedetomidine reversal agents, like the α_2_-receptor antagonist atipamezole, cause serious adverse effects at the large dosages required for effective reversal; they are not used clinically. Without reversal agents, emergence times from dexmedetomidine sedation are slow. In this study we tested the ability of low-dose atipamezole, in combination with caffeine, to reverse dexmedetomidine sedation. The low dose of atipamezole employed should not be associated with unwanted effects.

**Methods:**

Two different sedation protocols were employed. In the first protocol, a bolus of dexmedetomidine was rapidly applied and the drug was allowed to equilibrate for 10 min before rats received either saline (as control) or low-dose atipamezole with caffeine. Following this procedure, rats were placed on their backs. Emergence from sedation was the time for rats to recover their righting reflex and stand with 4 paws on the floor. A second sedation protocol simulated a pediatric magnetic resonance imaging (MRI) scan. Adult rats were sedated with dexmedetomidine for one hour followed by 30 min with both dexmedetomidine and propofol. At the end of 90 min, rats received either saline (control) or a combination of low-dose atipamezole, and caffeine. Recovery of the righting reflex was used as a proxy for emergence from sedation.

**Results:**

Emergence from sedation, the time for rats to recover their righting reflex, decreased by ~ 90% when using an atipamezole dose ~ 20 fold lower than manufacturer’s recommendation, supplemented with caffeine. Using an atipamezole dose ~ tenfold lower than recommended, with caffeine, emergence times decreased by ~ 97%. A different stimulant, forskolin, when tested, was as effective as caffeine. For the MRI simulation, emergence times were decreased by ~ 93% by low-dose atipamezole with caffeine.

**Conclusions:**

Low dose atipamezole with caffeine was effective at reversing dexmedetomidine sedation. Emergence was rapid and the rats regained not only their righting reflex but also their balance and their ability to carry out complex behaviors. These findings suggest that the combination of low dose atipamezole with caffeine may permit rapid clinical reversal of dexmedetomidine without unwanted effects.

## Background

Dexmedetomidine is an intravenous sedative administered during general anesthesia, as an adjunct in neurosurgery [1] and in intensive care where it reduces time to extubation and intensive care unit (ICU) time [2]. Dexmedetomidine is popular for procedural sedation in children [3]. Dexmedetomidine is a selective α_2_-adrenergic receptor agonist [3]. α_2_ receptors are G-protein coupled receptors linked to the activation of the G_i/o_ signaling pathway [4], which results in the inhibition of adenylate cyclase thereby reducing intracellular cAMP levels and strong inhibition of neuronal activity [5].

Most anesthetics are linked to neuronal apoptosis and cognitive deficits in animal studies which include non-human primates [6, 7]. There appears to be a time window when the developing nervous system is susceptible to common anesthetics including propofol, isoflurane, sevoflurane and ketamine. Dexmedetomidine is popular in children because it is not associated with apoptosis; rather it appears neuroprotective [8]. In the elderly, sedation with dexmedetomidine may reduce the incidence of post-op delirium when used with other anesthetics [9, 10]. Unfortunately, dexmedetomidine is associated with very long emergence times [11].

Atipamezole is a selective α_2_-adrenergic receptor competitive antagonist [12, 13]. Atipamezole is widely used in veterinary medicine to reverse dexmedetomidine [14] but not in humans due to unwanted symptoms, such as emesis, motor restlessness, and an increase in blood pressure (BP) [13, 15–17]. At high dosages (100 mg per test subject) mean arterial BP was elevated by > 20 mmHg [13]. In humans, effective reversal of dexmedetomidine sedation needed atipamezole dosages 40–100-fold higher than that of dexmedetomidine [18]; these doses are associated with the unwanted effects listed above.

Because dexmedetomidine activates α_2_ receptors and reduces intracellular [cAMP] levels, drugs that restore cAMP, may reverse the G_i/o_ mediated effects. Caffeine inhibits phosphodiesterase, thereby preventing the breakdown of cAMP, which elevates intracellular cAMP levels [19]. Forskolin is a stimulator of adenylate cyclase, which results in the elevation of intracellular cAMP [20]. Other stimulants that elevate [cAMP]_i_ may be equally effective.

Competitive antagonists, like atipamezole, shift agonist dose response curves along the abscissa to higher concentrations, thereby requiring a higher dose of agonist to produce a given response [21]. Our goal in this study was to repurpose atipamezole from a dexmedetomidine reversal agent, where only very high doses of the drug are effective, to one where a low dose of the drug was used to lighten sedation thereby allowing caffeine or forskolin to reverse the remaining sedative effects. Caffeine or forskolin decreased the dose of atipamezole that reversed dexmedetomidine. This strategy was successful in rats and awaits testing in humans.

## Materials and methods

### Ethics and animals

This study on rats was approved by The University of Chicago Institutional Animal Care and Use Committees (IACUCs) with protocol #42,437. In between experiments animals were cared for by University of Chicago veterinary staff. During experiments, heart rate (HR), respiratory rate (RR) and blood oxygen saturation (SpO_2_) were monitored with a Kent Scientific PhysioSuite. SpO_2_ was always > 92% and typically at 99% throughout the experiments showing that animals were not in distress. Blood pressure was measured with an IITC Inc NIBP Multi Channel Blood pressure System, using an inflatable cuff attached to the rat’s tail.

### Drugs

Caffeine (Sigma-Aldrich, St Louis, part # C0750-5G, Lot#SLBD0505V) was dissolved in sterile saline to a final concentration of 10 mg/ml, and rats were dosed intravenously to a final dose of 25 mg/kg. Sterile saline injection was used as vehicle control for caffeine.

Forskolin (Sigma-Aldritch) was dissolved in dimethyl sulfoxide (DMSO) to 5 μM and then used at 0.3 mg/kg. The DMSO-containing solution was placed in the I-V line and then flushed into the rats with 0.5 ml of saline.

Atipamezole (also called Antisedan) was manufactured by Zoetis Pharmaceuticals, Parsippany, NJ (#RXANTISEDAN-10). The same bottle was used for the entire study. The atipamezole dosages used in the studies outlined in the manuscript varied from 5 μg/kg to 20 μg/kg when supplemented with caffeine. Atipamezole was administered intravenously to the rats with sterile saline as the vehicle at 5 µg/ml.

Dexmedetomidine (Dexmedetomidine hydrochloride: NDC 16,729–239-93) was bought from Accord Healthcare, Durham, NC. The concentration of dexmedetomidine was 5 µg/ml in saline. The dexmedetomidine dose used in these studies was 10 μg/kg when it was applied as an intravenous bolus. In that case, drug injection was performed manually to ensure rapid perfusion (~ 30 s). In some experiments dexmedetomidine was applied via a pump (Medfusion 4000, Smith Medical ASD, Inc. St. Paul, MN) which took 5 min to deliver the full dose of 10 μg/ kg.

### Instrumentation and sedation

For these experiments, rats were placed in a gas-tight anesthesia chamber where they were exposed to 1.8% (~ 1.4 MAC) isoflurane (in 4 L/min, 2 Liters O_2_/2 L Air) for 12 min. During this time, the rats became unconscious and insensitive to tail pinch. Rats were then removed from the gas tight chamber and weighed. Anesthesia was maintained with 1.8% isoflurane in 2 L/ min, 1L O_2_/1 L Air) delivered via a nose cone. A 24 gauge intravenous (IV) catheter was inserted into a tail vein. After 20 min total time at 1.8% isoflurane, including anesthesia box and nose cone, isoflurane was decreased to 1.1% and allowed to equilibrate for 5 min.

Thirty-two female Adult Sprague Dawley rats (Charles River, Wilmington, MA), weighing 250–350 gm were used in the study. Eight male rats weighing 450–600 gm were also used, in a single experiment with atipamezole and caffeine. All rats were housed in the same room in the University of Chicago animal care unit and were cared for by facility staff. They were transported to the anesthesia room for multiple sedation sessions with at least five days in between sessions. At the completion of each anesthesia session, rats were transported back to their own home room. Rats were divided into groups of 8 for each set of experiments. All rats received drug injection or saline in a repeated measure design. Rats were never sedated more than 6 times. Because there was a small variation in responsiveness to sedation between groups of rats, all rats served as their own controls. When the same rats were tested multiple times, the responses were reproducible. All experiments were performed during the daytime and at room temperature of 22–27 ^◦^C. While on a nose cone and throughout the study rats were placed on a heating pad at 25 °C. At the conclusions of the study, rats were euthanized by the animal facility staffs using CO_2_ overdose, followed by decapitation.

The entire study was completed in female rats. The solitary group of 8 male rats was employed to ensure that rapid emergence from dexmedetomidine sedation after atipamezole and caffeine injection was not sex dependent. Adult male rats were much larger than were the female rats (males range 450–600 g vs females 250–350) at the same developmental stage.

### Experimental protocol

Two different sedation protocols were employed in this study.

**Fig. 1:**
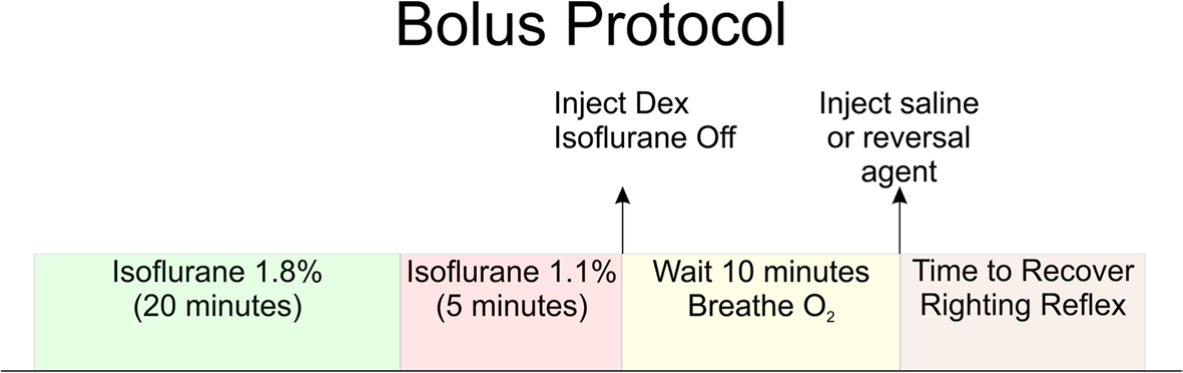
Experimental protocol for bolus injections of dexmedetomidine and reversal. Bolus injections were followed by a 10-min pause for dexmedetomidine to equilibrate. The 10-min waiting period also allows for washout of residual isoflurane. At the end of the 10-min equilibration period, the dexmedetomidine dosage should be approximately what was injected. At this time the rats received an injection of either saline, atipamezole, caffeine or atipamezole with caffeine

For the first set of experiments, a bolus of dexmedetomidine was delivered manually, in ~ 30 s, via a syringe to the rats (see Fig. 1). In these experiments, the rats were placed in an anesthesia box, 1.8% isoflurane, for 12 min. After they were unconscious an intravenous (IV) catheter was inserted. At 20 min, the isoflurane was reduced to 1.1% for 5 min. At 25 min the rats received a bolus of dexmedetomidine delivered manually via a syringe directly to the IV catheter over 30 s. Immediately afterward, isoflurane was ended, and the rats breathed O_2_/Air. This was followed by a 10-min period to allow the dexmedetomidine to equilibrate. We then administered the reversal drugs or saline. The half-life of dexmedetomidine elimination in rats is ~ 65 min [22]. In this protocol, dexmedetomidine levels should not have changed too much during the 10 min waiting period, from what was injected. After administration of the reversal agents or saline control the IV catheter was removed, and the rats were placed in a cage on their backs. The time to emerge from sedation was defined as the time required for the rats to flip over and stand with 4 paws on the bottom of the cage (also referred to as recovery of righting reflex—RORR).

Dexmedetomidine is commonly used to sedate children to prevent movement artifacts in medical imaging procedures like MRI [3, 23]. Dexmedetomidine alone can prevent movement for brief periods, but not reliably for sedation durations > 1 h. Clinically, longer procedures are often managed with dexmedetomidine and with a low dose of second sedative, like propofol, midazolam or thiopental, added later or after any movement is seen [23, 24]. To mimic such procedure in rats, a bolus of dexmedetomidine (10 μg/kg delivered over 5 min via a pump) was followed by an 85-min continuous infusion of dexmedetomidine (10 μg/kg/hr). At 55 min, a bolus of propofol was administered (4 mg/kg over 5 min) followed by a continuous infusion of propofol at a rate of 200 μg/kg/min for the next 30 min. For the last 35 min dexmedetomidine and propofol were given together. This dose of propofol alone, was not able to produce a loss of righting reflex in rats. The second lumen of a Y shaped microcatheter (Baxter Healthcare Corp, Deerfield, IL) allowed us to use a different pump for propofol. As a result, the two drugs, dexmedetomidine and propofol, did not mix until they reached the small IV catheter so changing the infusion rate of one drug did not affect the other.

### Statistical analysis

**Fig. 2:**
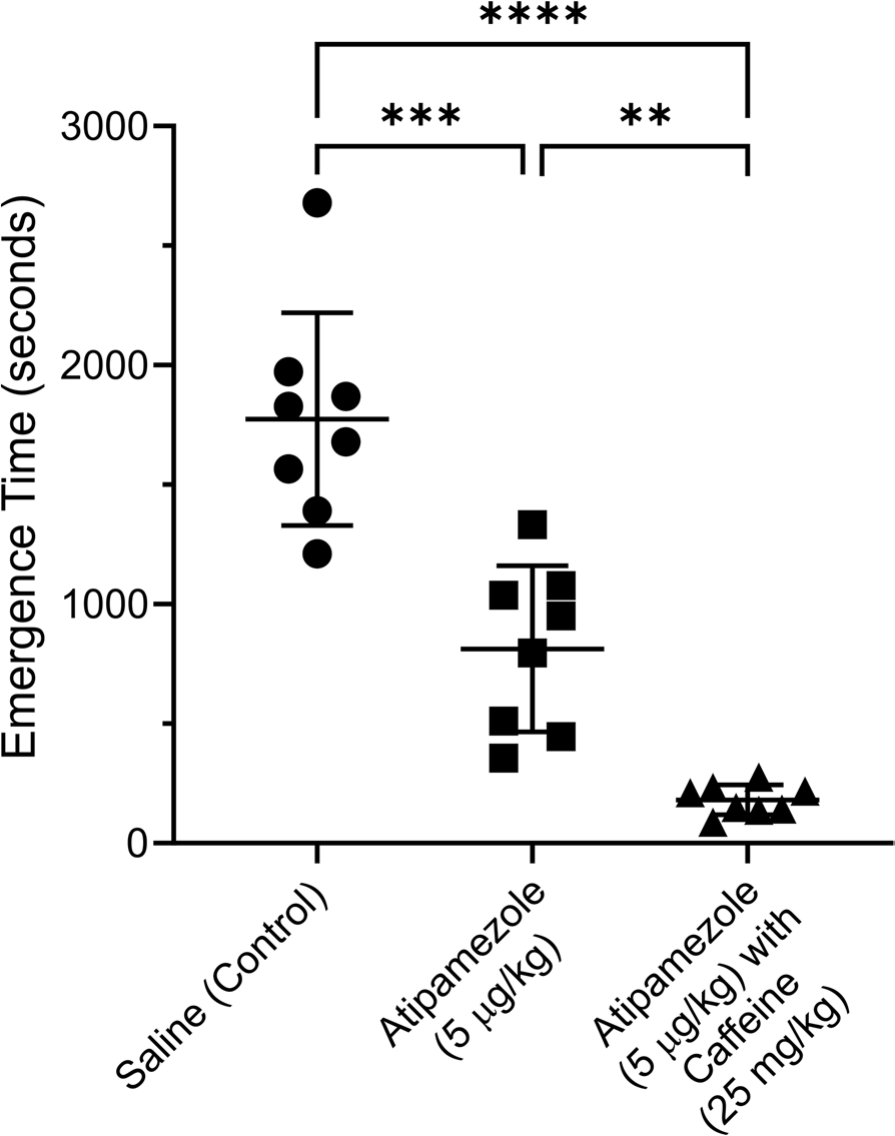
The combination of atipamezole with caffeine dramatically accelerated emergence from dexmedetomidine sedation compared to that produced by saline or atipamezole by itself. A group of 8 female rats were exposed to 3 deep sedation sessions using bolus injections of dexmedetomidine (10 µg/kg), a week apart. The protocol used is described in Fig. 1. At the end of each session the rats received a bolus injection of either saline or atipamezole (5 μg/kg) or atipamezole (5 μg/kg) & caffeine (25 mg/kg). The order of the drug injections was randomized. Rats were placed on their backs in a waking box, and the time for the rats to recover their righting reflex was recorded. This time is plotted in the figure as the emergence time. The figure plots the time to emerge from sedation for rats receiving saline (leftmost group) or atipamezole (middle group) or atipamezole with caffeine (rightmost group). A one-way repeated measures ANOVA revealed that there was a statistically significant difference in emergence times between at least two groups (F(2,14)) = 63.9, *p* < 0.0001. Tukey’s HSD Test for multiple comparisons found that the mean value for emergence from sedation was different for saline (control) vs atipamezole (*p* = 0.0009, 95% C.I. = [522 to 1401]); for saline (control) vs atipamezole with caffeine (*p* < 0.0001, 95% C.I. = [1129, 2057]); for atipamezole vs atipamezole with caffeine (*p* = 0.0023, 95% C.I. = [292 to 972]).RORR Times (in seconds): Female rats shown in Fig. 2: Saline (control): 1210, 1869, 1828, 1390, 1973, 1679, 2679, 1565, Atipamezole (5 μg/kg): 510, 446, 1332, 950, 1037, 795, 1078, 356, Atipamezole (5 μg/kg) with Caffeine (25 mg/kg): 142, 87, 232, 137, 276, 210, 149, 217. Male rats—Data presented in the Results Section: Saline (control): 2305, 2941, 5045, 2200, 1355, 2012, 1921, 3862; Atipamezole (5 µg/kg) & Caffeine (25 mg/kg): 79, 100, 151, 30, 25, 55, 163, 347

**Fig. 3:**
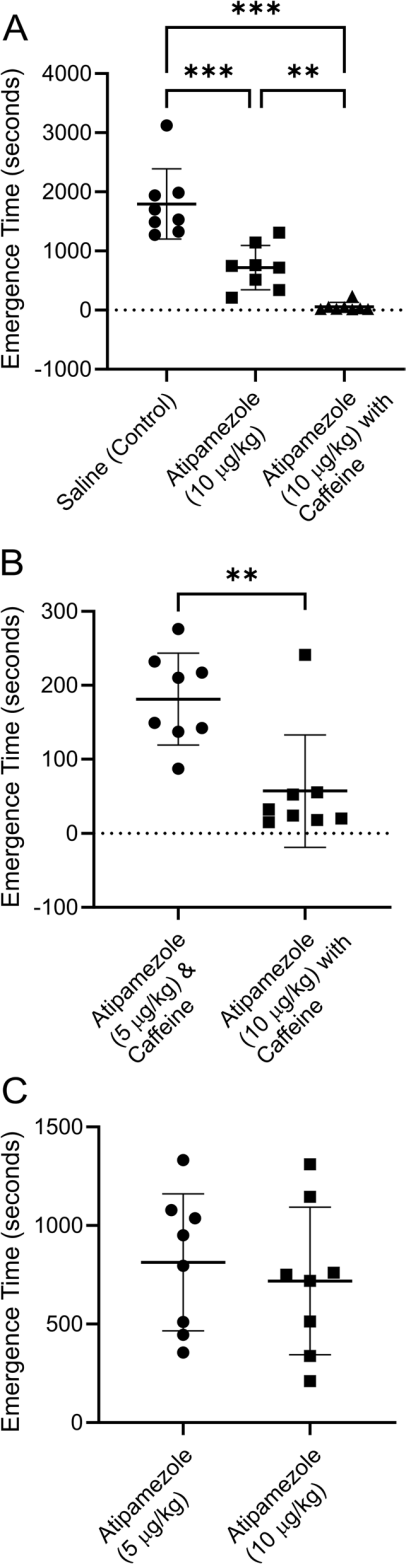
A higher dose of atipamezole and caffeine (see Fig. 2) accelerated emergence from dexmedetomidine sedation even more dramatically. Atipamezole by itself was not as effective as atipamezole with caffeine. The same group of 8 rats shown in Fig. 1 were exposed to 3 deep sedation sessions using bolus injections of dexmedetomidine (10 µg/kg), a week apart (protocol shown in supplementary material). At the end of each session the rats received a bolus injection of either saline or atipamezole (10 μg/kg) or atipamezole (10 μg/kg) with caffeine (25 mg/kg). The order of the drug injections was randomized. Rats were placed on their backs in a waking box, and the time for the rats to recover their righting reflex was recorded. This time is plotted in the figure as the emergence time. **A**, plots the time to emerge from sedation for rats receiving saline (leftmost group) or atipamezole (middle group) or atipamezole with caffeine (rightmost group). Using a repeated measures ANOVA (Tukey’s HSD Test for multiple comparisons) the mean value for emergence from sedation was significantly different for saline (control) vs atipamezole (*p* = 0.0003, 95% C.I. = [669 to 1485]); between saline (control) vs atipamezole with caffeine (*p* = 0.0002, 95% C.I. = [1096, 2381]); between atipamezole vs atipamezole with caffeine (*p* = 0.006, 95% C.I. = [245 to 1078]). **B**, Compares emergence times for 5 μg/kg atipamezole with caffeine with 10 μg/kg atipamezole with caffeine. The difference was significant (*p* = .002; paired T-test; *n* = 8). **C**, Compares emergence times for 5 μg/kg atipamezole with 10 μg/kg atipamezole. The difference was not significant (*p* = 0.62; paired T-test, *n* = 8). RORR Times (in seconds): Saline (control): 1938, 1532, 1490, 1273, 3121, 1700, 1985, 1325, Atipamezole (10 μg/kg): 750, 1145, 513, 210, 1311, 719, 760, 338, Atipamezole (10 μg/kg) with Caffeine (25 mg/kg): 15, 24, 241, 18, 32, 52, 20, 55

In this study the threshold for statistical significance was set to 0.05. The statistical test used to analyze each data set is described in the figure legends. If three or more comparisons were needed within a group of animals a repeated measures analysis of variance (RM-ANOVA) with Tukey’s multiple comparisons post-hoc test was employed. Data were tested for normality (D'Agostino-Pearson test). When only 2 conditions were tested, a T-test was employed. Data were analyzed and graphed using GraphPad Prism 9 software. A software tool, G*Power, allowed us to find appropriate sample population sizes. Using the data in Figs. 2 and 3, G*Power suggested minimum population sizes of either 3 or 4 rats were sufficient for these experiments given the variance in the data and the size of the effect.

The experiments shown in this manuscript were done in an unblinded manner.

## Results

At no time in these studies did animals display behaviors consistent with pain.

Competitive antagonists shift dose response curves along the abscissa towards higher concentrations. In the presence of even a low dose of a competitive antagonist, higher agonist concentrations are required to produce an equivalent effect [21]. Caffeine has the ability to reverse modest levels of sedation [25]. The goal of the current study was to determine whether a low dose of atipamezole, one which should not be associated with unwanted effects, could lessen deep sedation to a level that caffeine could reverse the remaining sedation.

For the protocol shown in Fig. 1, the rats were exposed to isoflurane for 20 min while an I-V was inserted (see Methods). The rats received a bolus of dexmedetomidine delivered manually via a syringe directly to the IV catheter over 30 s and immediately afterward, the isoflurane was stopped, and the rats breathed an O_2_/Air mixture (50%/ 50%, see methods). Next, a 10-min waiting period allowed the dexmedetomidine to equilibrate and the isoflurane to wash out. The reversal drugs or saline were then administered via the I-V. The IV catheter was removed, and the rats were placed in a cage on their backs. The time to emerge from sedation was defined as the time required for the rats to flip over and stand with 4 paws on the bottom of the cage (also referred to as recovery of righting reflex—RORR). The half-life for dexmedetomidine elimination is thought to be ~ 65 min in rodents [22]. For this protocol, because there was only 10 min for metabolism to take place before reversal agents were administered, we assumed that the dexmedetomidine levels in the rats were similar to those injected. This assumption needs to be verified in future studies by direct measurement of dexmedetomidine.

Atipamezole supplemented with caffeine accelerated emergence from dexmedetomidine sedation. A dexmedetomidine bolus (10 μg/kg) caused a deep sedation in a group of 8 rats. Each rat was sedated 3 times with each sedation session at least a week apart. After injection of saline (control), rats regained their righting reflex very slowly (1774 ± 445,mean ± SD seconds, *n* = 8), as shown in Fig. 2. In the same group of rats, injection of atipamezole alone (5 μg/kg) reduced the time by about half (813 ± 347 s, *n* = 8). Administration of atipamezole (5 μg/kg) with caffeine (25 mg/kg) reduced the emergence time by ~ 90% (181 ± 62 s, *n* = 8). Emergence in saline injected rats was significantly different than in rats injected with atipamezole (*p* = 0.0009; one-way repeated measures ANOVA, *n* = 8) or those injected with atipamezole & caffeine (*p* < 0.0001, *n* = 8). Atipamezole by itself was significantly different than atipamezole & caffeine (*p* = 0.0023, *n* = 8).

For the 10 μg/kg dexmedetomidine tested in Fig. 2, 100 μg/kg is the manufacturer’s recommended dose for reversal, when atipamezole is used by itself. The dosage used to generate the data in Fig. 2 was 20-fold lower. Atipamezole and caffeine used together resulted in rapid emergence from dexmedetomidine sedation.

A solitary group of 8 male rats was tested in the same manner as the female rats. After a bolus of 10 µg/kg of dexmedetomidine, male rats emerged slowly from sedation (2705 ± 1207 s, *n* = 8) after saline (control) injection. This was not different than the emergence time shown in Fig. 2 for females (*p* = 0.08, unpaired t-test, *n* = 8). Atipamezole (5 µg/kg) with caffeine (25 mg/kg) dramatically reduced emergence time in male rats just like it did in female rats (19 ± 105 s; *p* = 0.0004; paired t-test, *n* = 8, df = 7, t = 6.375). Neither caffeine nor atipamezole alone were tested in the male rats.

Figure 3A repeats the experiment shown in Fig. 2, with the same group of 8 rats, except that a higher dose of atipamezole was employed. The two-fold higher dose of atipamezole was more effective. On average, emergence was slow (1796 ± 595 s, *n* = 8), after injection of saline (control). Injection of atipamezole (10 μg/kg) reduced the emergence time by more than half (718 ± 374 s, *n* = 8). The combination of atipamezole with caffeine reduced the emergence time by ~ 97% (57 ± 76 s, *n* = 8). Emergence in saline injected rats was significantly different than in rats injected with atipamezole (*p* = 0.0003; repeated measures ANOVA) or those injected with atipamezole with caffeine (*p* = 0.0002). Atipamezole by itself was significantly different than atipamezole with caffeine (*p* = 0.0056).

The dosage of atipamezole employed was tenfold lower than the recommended dosage for rats. When used in combination with caffeine, reversal was very rapid. In fact, some of the rats recovered their righting reflex during the drug injections.

Figure 3B compares time to emergence from sedation when 5 μg/kg atipamezole was employed with caffeine with that when 10 μg/kg atipamezole was paired with caffeine. The difference was significant (*p* = 0.002; paired T-test, *n* = 8). The large standard deviation in the data is due to the single animal that emerged relatively slowly from sedation when given 10 μg/kg atipamezole with caffeine.

Figure 3C shows that when atipamezole was used as the sole reversal agent there was no difference in time to emergence between 5 μg/kg atipamezole and 10 μg/kg atipamezole (*p* = 0.62; paired T-test, *n* = 8).

**Fig. 4:**
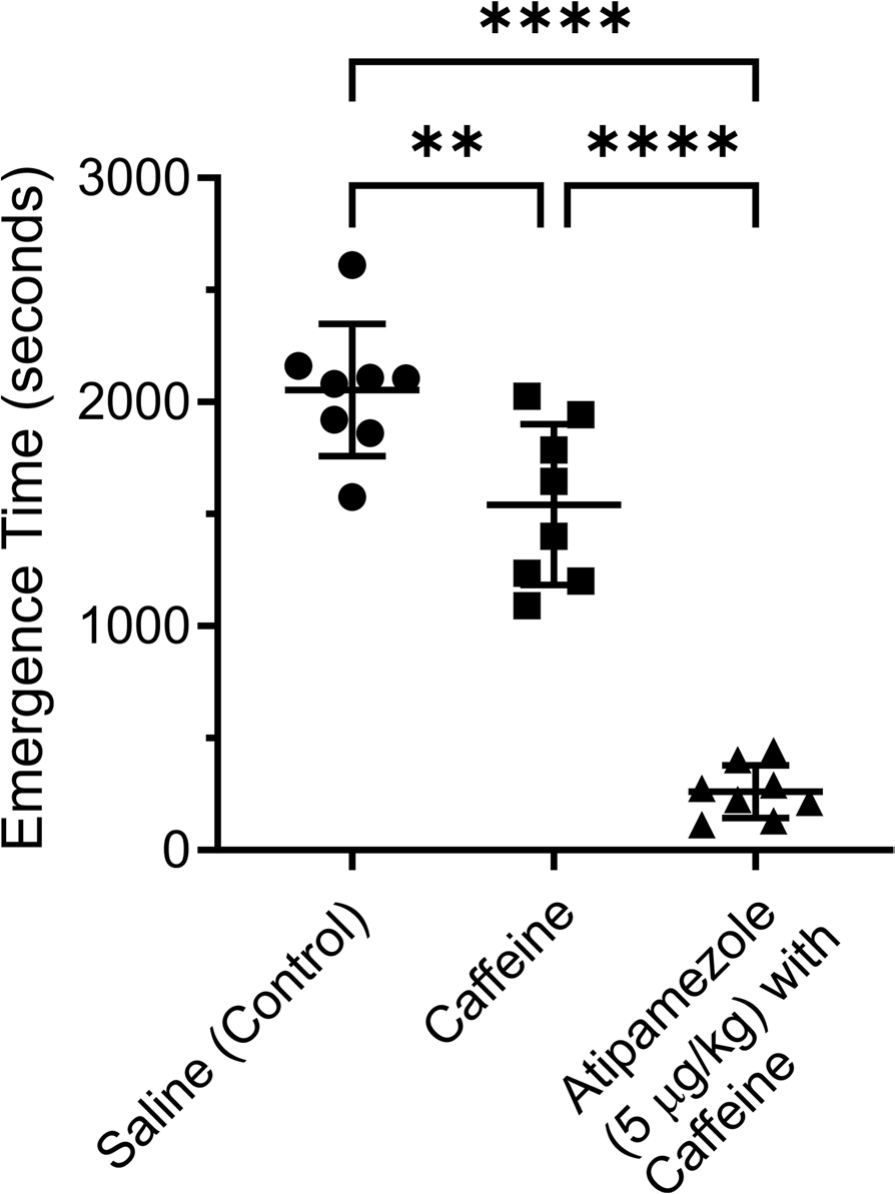
Caffeine by itself was not effective at reversing dexmedetomidine sedation. A group of 8 rats were exposed to 3 deep sedation sessions using bolus injections of dexmedetomidine, a week apart (protocol shown in supplementary material). At the end of each session the rats received a bolus injection of either saline or caffeine (25 mg/kg) or atipamezole (5 μg/kg) with caffeine (25 mg/kg). The order of the drug injections was randomized. Rats were placed on their backs in a waking box, and the time for the rats to recover their righting reflex was recorded. This time is plotted in the figure as the emergence time. The figure plots the time to emerge from sedation for rats receiving saline (leftmost group) or caffeine (middle group) or atipamezole with caffeine (rightmost group). Emergence from sedation was significantly different for saline (control) vs caffeine (repeated measures ANOVA, *p* = 0.0027, 95% C.I. = [230 to 793]); between saline (control) vs atipamezole with caffeine (*p* < 0.0001, 95% C.I. = [1539, 2044]); between caffeine vs atipamezole with caffeine (*p* < 0.0001, 95% C.I. = [968 to 1592]). RORR Times (in seconds): Saline (control): 1922, 1861, 1577, 208, 2109, 2105, 2162, 2610, Caffeine (25 mg/kg): 1787, 1092, 1237, 1401, 1646, 1202, 1944, 2027 Atipamezole (5 μg/ kg) with Caffeine (25 mg/kg): 403, 213, 130, 292, 114, 225, 276, 443

Figure 4 shows data from a different group of rats using the same bolus protocol. On average emergence from dexmedetomidine sedation was slow (2053 ± 295 s (*n* = 8)), after saline (control) injection. Injection of caffeine (25 mg/kg) reduced the emergence time by about a quarter (1542 ± 35 s (*n* = 8)). The combination of atipamezole (5 µg/kg) with caffeine (25 mg/kg) dramatically reduced emergence time (262 ± 117 s (*n* = 8)). Emergence in saline injected rats was significantly different than in rats injected with caffeine (*p* = 0.0027) or those injected with atipamezole with caffeine (*p* < 0.0001). Caffeine by itself was significantly different than atipamezole with caffeine (*p* < 0.0001).

**Fig. 5:**
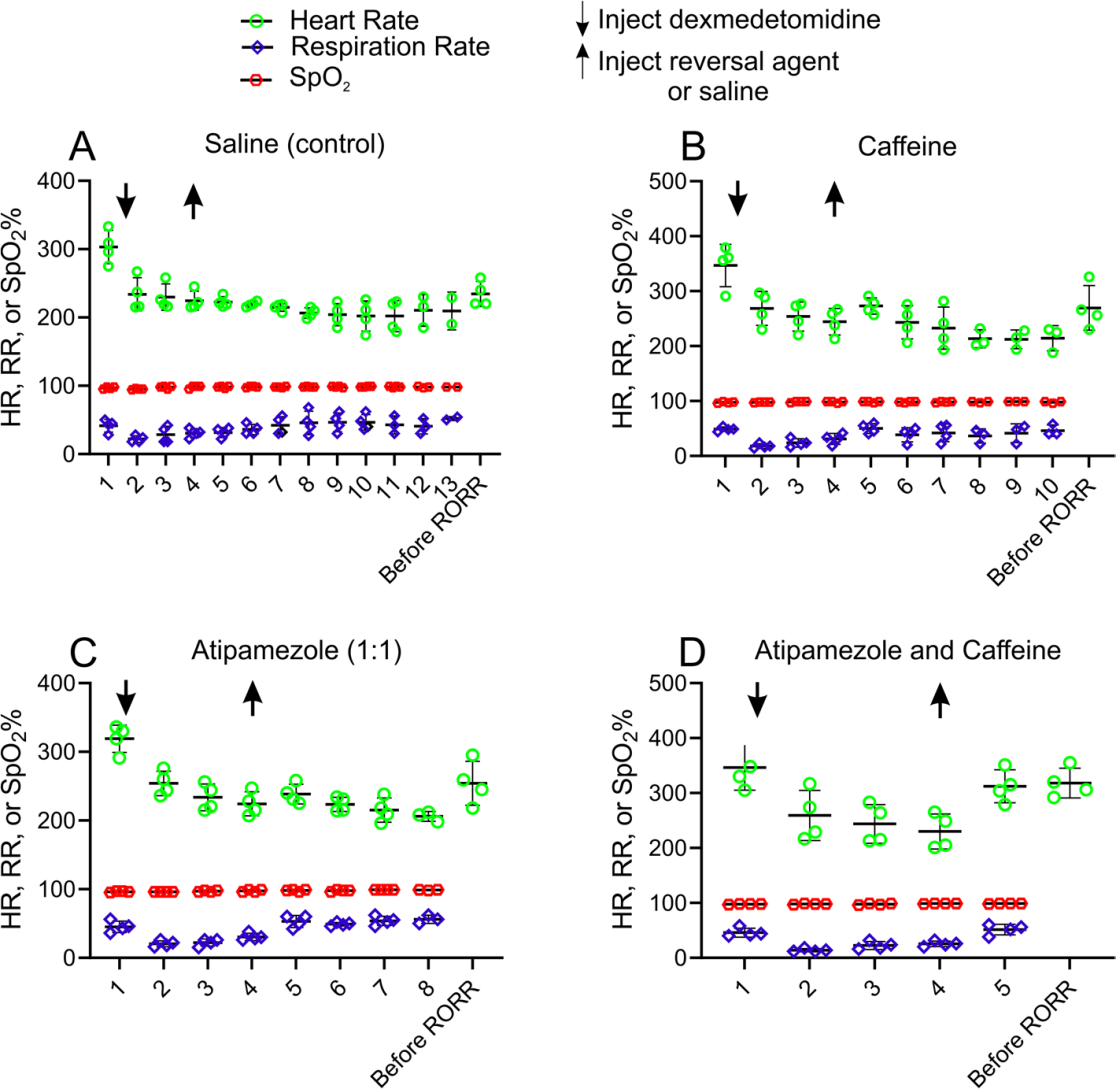
Rapid recovery of vital signs after atipamezole and caffeine (25 mg/kg). Vital sign comparisons were analyzed at different times points (1.1% isoflurane, 30 s post dexmedetomidine, 5 min post dexmedetomidine, 10 min post dexmedetomidine, 30 s post saline or reversal, 5 min post saline or reversal, 10 min post saline or reversal and before RORR) in each session using an one-way repeated measures ANOVA with Tukey’s HSD Test for multiple comparisons (*n* = 4). The vital signs at the time point of 1.1% isoflurane (1) was used as the control. In Fig. 5A (saline session), the HR at time point (1) was significantly different from each of those at time points (2–10) (each adjusted *p* < 0.05) and was not significantly different from one before RORR (adjusted *p* < 0.11). The RR at time point (1) was not significantly different from each of those at time points (2–10) (each adjusted *p* > 0.05). In Fig. 5B (caffeine session), the HR at time point (1) was significantly different from each of those at time points (2, 3, 4, 6 and 7) (each adjusted *p* < 0.05) and was not significantly different from those at the time point 30 s post caffeine (5) (*p* < 0.11) and the one before RORR (adjusted *p* = 0.05). The RR at time point (1) was significantly different from the one at time point 30 s post dexmedetomidine (2) (adjusted *p* < 0.03) and not significantly different from each of those at time points (3–7) (each adjusted *p* > 0.05). In Fig. 5C (atipamezole session), the HR at time point (1) was significantly different from each of those at time points (2–7) (each adjusted *p* < 0.05) and was not significantly different from the one before RORR (adjusted *p* = 0.10). The RR at time point (1) was not significantly different from each of those at time points (2–7) (each adjusted *p* > 0.05). In D (atipamezole and caffeine session), the HR at time point (1) was significantly different from each of those at time points (2, 3 and 4) (each adjusted *p* < 0.05) and was not significantly different from those at the time point 30 s post caffeine (5) (*p* = 0.05) and the one before RORR (adjusted *p* = 0.13). The RR at time point (1) was significantly different from the one at time point 30 s post dexmedetomidine (2) (adjusted *p* < 0.05) and not significantly different from each of those at time points (3–5) (each adjusted *p* > 0.05). Times to RORR varied from rats to rats in each session, (saline, 2324 ± 369 s; caffeine, 1444 ± 414 s; atipamezole, 951 ± 242 s; atipamezole with caffeine, 42 ± 8 s, *n* = 4). Since rats emerged at different time points within the session and between sessions, ANOVA only analyzed at the available time points without missing data in each session. Note that HR was significantly reduced after the bolus of dexmedetomidine in each session. HR before RORR returned to near the level recorded at pre-dexmedetomidine with 1.1% isoflurane in all sessions. The time point before RORR occurred much earlier in atipamezole with caffeine session (**D**) than those in saline (**A**), caffeine (**B**) and atipamezole (**C**) sessions

Figure 5 plots vital signs for a group of 4 female rats exposed to a bolus of dexmedetomidine (10 µg/kg). Ten minutes after the bolus of dexmedetomidine and washout of isoflurane, saline or one of the following reversals was given; (A) saline (0.5 ml), (B) caffeine (25 mg/kg), (C) atipamezole (10 µg/kg) or (D) atipamezole (10 µg/kg) with caffeine (25 mg/kg) in one of those 4 sessions. Only atipamezole with caffeine rapidly restored vital signs to their pre-dexmedetomidine levels. Atipamezole with caffeine rapidly reversed the bradycardia produced by dexmedetomidine, while saline (control), atipamezole or caffeine alone did not. Vital signs, HR, RR and SpO_2_, were recorded at different time points for each session; (1) 1.1% isoflurane, (2), 30 s post dexmedetomidine bolus, (3) 5 min post dexmedetomidine bolus, (4) 10 min post dexmedetomidine bolus, (5) 30 s post saline or one of the reversals, (6) 5 min post saline or one of the reversals, (7) 10 min post saline or one of the reversals, (8) 15 min post saline or one of the reversals, (9) 20 min post saline or one of the reversals, (10) 25 min post saline or one of the reversals, and before RORR. The “down” (dexmedetomidine) and “up” (saline or reversal drug) arrows represent the time points when the reversal drugs were administered. The data show that Dex produced a significant reduction in HR and RR but that these vital signs were soon restored following the administration of low-dose atipamezole and caffeine.

**Fig. 6:**
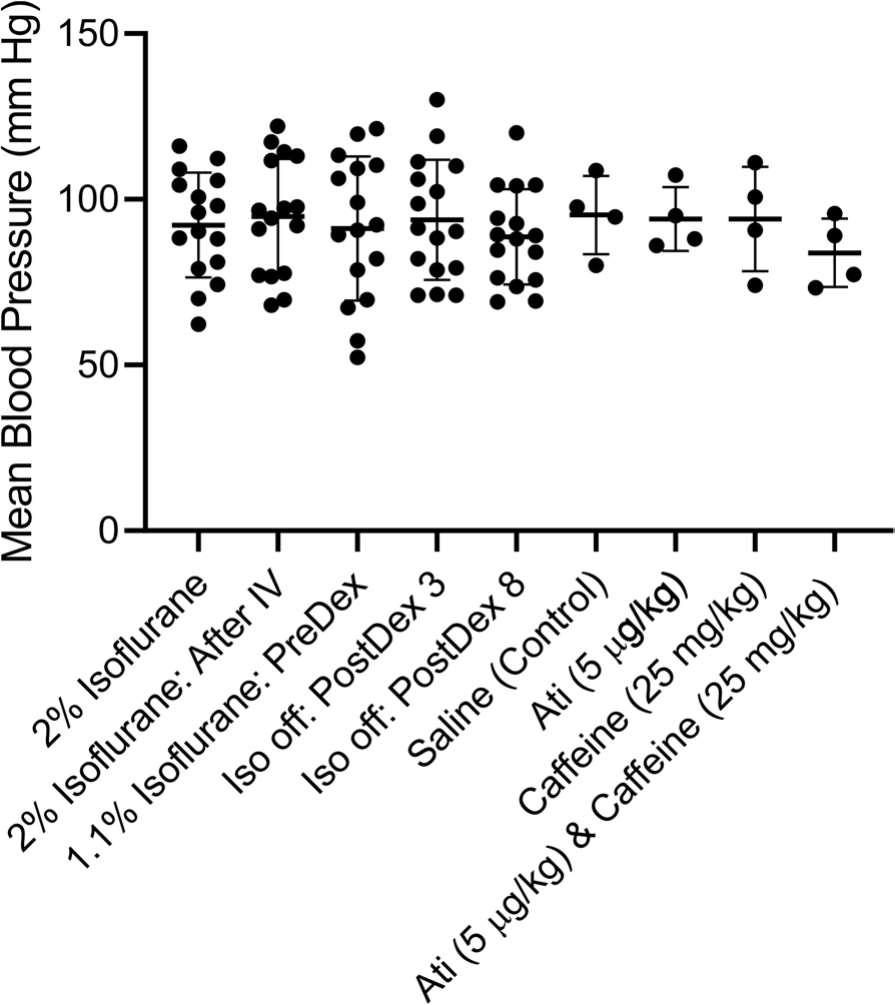
No change in mean blood pressure was observed after infusion of dexmedetomidine or the reversal agents. Blood pressure was measured with an inflatable tail cuff measurement system at different times. For these studies a group of 8 rats were used. Each rat received 2 sessions of dexmedetomidine sedation, for a total of 16 sessions. Saline, atipamezole, caffeine and atipamezole & caffeine were tested (4 sessions each) 10 min after dexmedetomidine injection. Blood pressure was measured at the following times: a) while rats were receiving 2% isoflurane. b) immediately after insertion of the I-V in the continued presence of 2% isoflurane. c) 3 min after turning the isoflurane level to 1.1% immediately prior to injecting dexmedetomidine (10 μg/kg). d) 3 min after dexmedetomidine injection with the rats breathing and O_2_/air mixture (no isoflurane). e) 8 min after dexmedetomidine injection with the rats breathing and O_2_/air mixture (no isoflurane). A final measurement was made 1 min following injection of either saline, or atipamezole (5 μg/kg), or caffeine (25 mg/kg), or atipamezole (5 μg/kg) & caffeine (25 mg/kg). (*n* = 4 each). Mean blood pressure values: 2% Isoflurane: 81, 101, 106, 90, 116, 79, 62, 74, 74, 88,109, 104, 88, 70, 98, 112, 96, 2% Isoflurane—After IV: 77, 97, 117, 97, 112, 92, 68, 78, 91, 114, 113, 77, 70, 94, 122, 98, 1.1% Isoflurane: 82, 57, 110, 91, 109, 92, 67, 99, 52, 106, 113, 79, 70, 120, 121, 89, 3 min after dexmedetomidine injection – isoflurane off: 82, 130, 99, 88, 111, 71, 71, 90, 119, 110, 106, 71, 79, 91, 79, 102, 8 min after dexmedetomidine injection – isoflurane off: 76, 120, 89, 84, 104, 76, 69, 85, 104, 104, 94, 74, 69, 88, 89, 93, 1 min after saline injection: 80, 109, 98, 95, 1 min after atipamezole (5 μg/kg) injection: 95, 86, 88, 107, 1 min after caffeine (25 mg/kg) injection: 101, 91, 74, 111, 1 min after atipamezole (5 μg/kg) & caffeine (25 mg/kg) injection: 77, 73, 96, 89

Figure 6 shows that blood pressure was not affected by the caffeine alone, atipamezole alone or the combination of atipamezole with caffeine. Using an ANOVA analysis, there was no significant different in blood pressure detected after dexmedetomidine injection or after any of the reversal drugs.

**Fig. 7:**
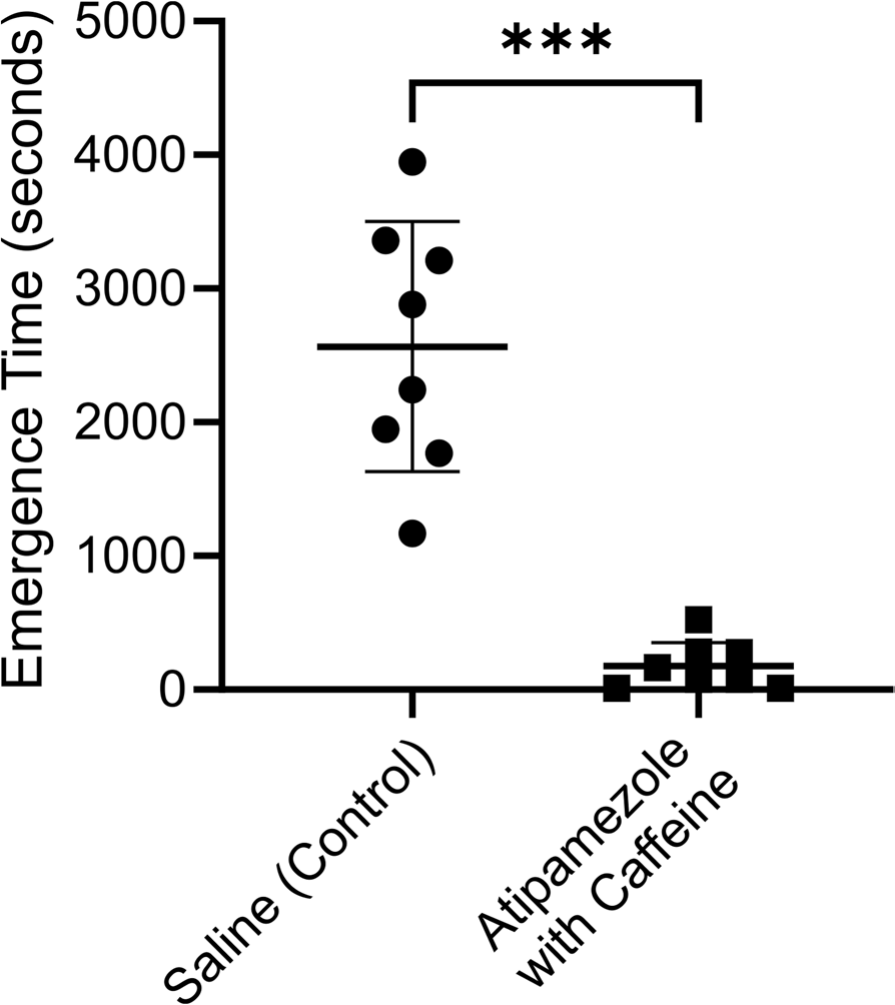
The combination of atipamezole with caffeine dramatically accelerated emergence from a simulated pediatric MRI sedation procedure. The same group of 8 rats were exposed to two sedation sessions, a week apart. At the end of one session the rats received a bolus injection of saline and in the other atipamezole (6 µg/kg) with caffeine (25 mg/kg). The order of the drug injections was randomized. Rats were placed on their backs in a waking box, and the time for the rats to recover their righting reflex was recorded. This time is plotted in the figure as the emergence time. The figure plots the time to emerge from sedation for rats receiving saline (leftmost group) or the same rats receiving atipamezole with caffeine (rightmost group). There was ~ 93% decrease in Emergence Time. The difference was significant (*p* = 0.0001; paired t-test). RORR Times (in seconds): Saline (control): 1170, 1948, 1769, 3947, 2244, 2880, 3211, 3361, Atipamezole (6 μg/kg) with Caffeine (25 mg/kg): 75, 12, 12, 77, 280, 165, 522, 275

Dexmedetomidine is commonly used to sedate children to prevent movement artifacts in medical imaging procedures like MRI [3, 23]. Dexmedetomidine by itself can prevent movement for brief periods, but not reliably for sedation durations > 1 h. Clinically, longer procedures are often managed with dexmedetomidine and with a low dose of second sedative, like propofol, midazolam or thiopental, added later or after any movement is seen [23, 24]. For this simulated pediatric MRI session, dexmedetomidine was perfused into the rats for 90 min, with propofol infused alongside the dexmedetomidine for the final 35 min (see Methods for further details). Figure 7 plots emergence data from the simulated pediatric MRI session. Each rat was sedated twice. In one sedation session, the rat received an injection of saline (control) and in the other session an injection of atipamezole (6 μg/kg) with caffeine (25 mg/kg). The order of the injections was randomized. For saline injected rats, emergence time was slow (2566 ± 935 s, *n* = 8) while for rats injected with atipamezole with caffeine emergence was reduced by ~ 93% (177 ± 175 s, *n* = 8). This difference was significant (*p* = 0.0001; paired t-test).

**Fig. 8:**
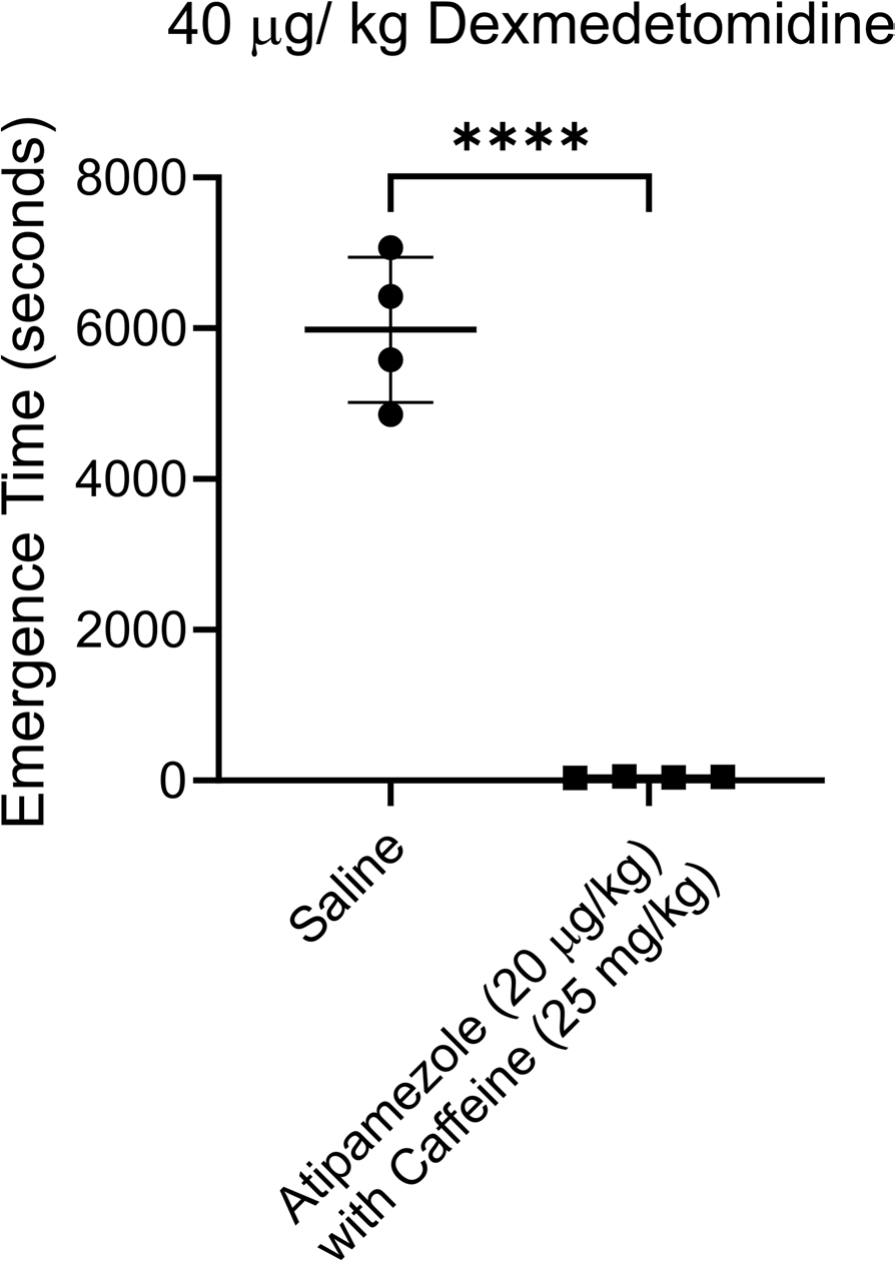
Atipamezole and caffeine effectively reversed high dose (40 µg/kg) dexmedetomidine sedation. A higher dose of atipamezole (20 µg) with caffeine (25 mg/ kg) dramatically accelerated emergence from the deep sedation produced by this high dose of dexmedetomidine. Two distinct groups of 4 rats were exposed to a sedation session using bolus injections of dexmedetomidine. One group received an injection of saline while the other group received atipamezole (20 µg/kg) with caffeine (25 mg/kg). Rats were placed on their backs in a waking box, and the time for the rats to recover their righting reflex was recorded. This time is plotted in the figure as the emergence time. Emergence in saline injected rats was significantly different than in rats injected with atipamezole & caffeine (*p* < 0.0001; *n* = 4, unpaired t-test). RORR Times (in seconds): Saline (control): 4857, 7067, 6420, 5580, Atipamezole (20 µg/kg) with Caffeine (25 mg/kg): 42, 39, 58, 47

Atipamezole with caffeine was equally effective at reversing high dosages of dexmedetomidine (Fig. 8). Emergence from 40 μg/ kg bolus of dexmedetomidine took < 1 min when atipamezole (20 µg/kg) and caffeine (25 mg/kg) were injected (46.5 ± 8 s: *n* = 4), while emergence took ~ 100 min when saline was injected (5981 ± 965.5 s: *n* = 4). The difference was significant (*p* < 0.0001, unpaired t-test).

**Fig. 9:**
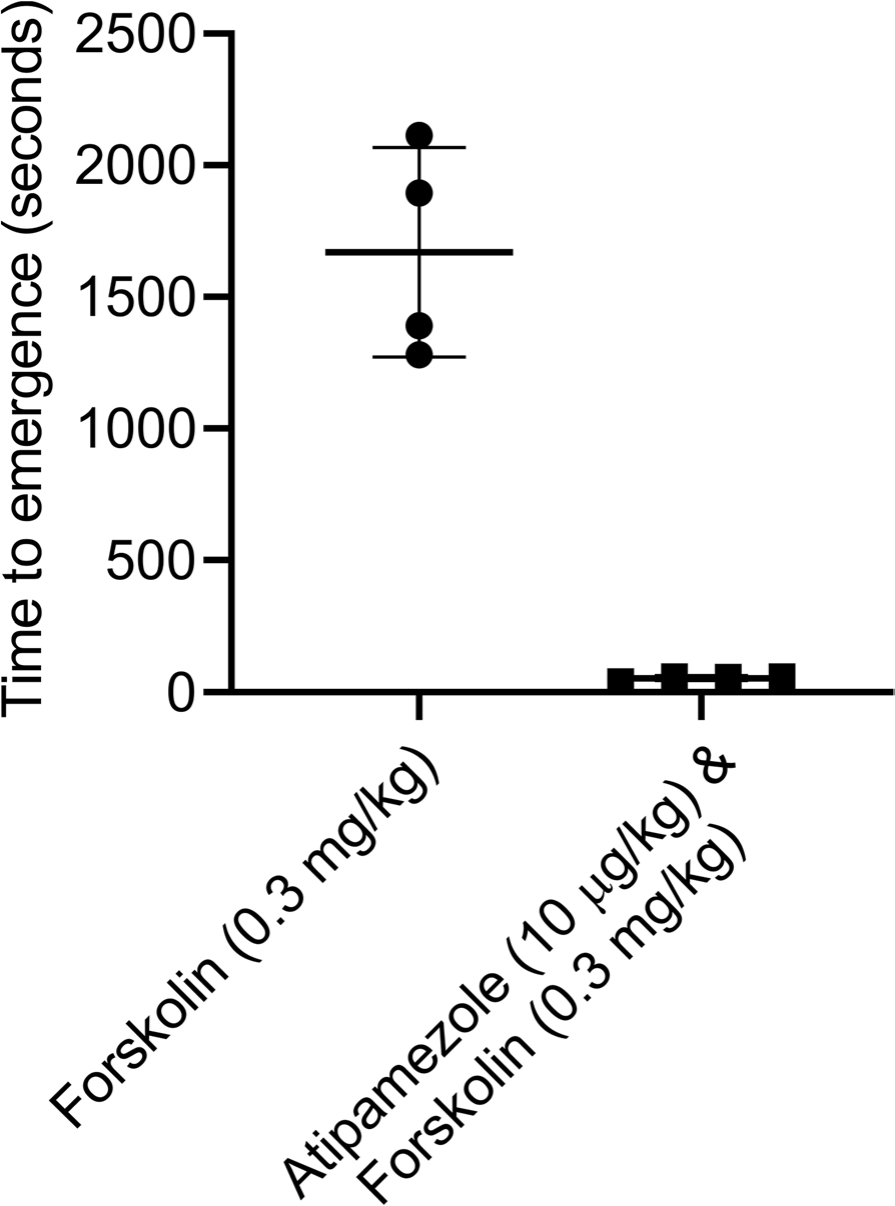
The combination of atipamezole with forskolin dramatically accelerated emergence from the deep sedation compared to that produced by forskolin alone. Two groups of 4 rats were exposed to a sedation session using a bolus injection of dexmedetomidine. At the end of each session the rats received a bolus injection of either forskolin (0.3 mg/kg) or atipamezole (10 µg/kg) with forskolin (0.3 mg/kg). The figure plots the time to emerge from sedation for rats receiving forskolin alone (leftmost group) or atipamezole with forskolin (rightmost group). Emergence in forskolin injected rats was significantly different than in rats injected with atipamezole with forskolin (*p* = 0.0002; unpaired t-test, *n* = 4). RORR Times (in seconds): Forskolin (0.3 mg/kg) alone: 1281, 1391, 1894, 2113, Atipamezole (10 µg/ kg) with forskolin (0.3 mg/kg): 41, 59, 59, 57

Forskolin, a drug that stimulates adenylate cyclase thereby elevating [cAMP]_i_ was effective at accelerating emergence from dexmedetomidine sedation, when used in combination with low-dose atipamezole (Fig. 9). Emergence from dexmedetomidine sedation took 1670 ± 398.2 s (*n* = 4) when forskolin alone was injected (0.3 mg/kg), but the combination of atipamezole (10 μg/kg) with forskolin (0.3 mg/kg) decreased emergence to 54 ± 8.7 s (*n* = 4). This ~ 97% reduction in emergence time was significant (*p* = 0.0002, unpaired t-test, *n* = 4). Forskolin was as effective as caffeine.

High-dose atipamezole (100 μg/ kg) by itself was as effective as low dose atipamezole (10 μg/kg) with caffeine (25 mg/kg) at reversing dexmedetomidine sedation. Emergence from a 10 μg/ kg bolus of dexmedetomidine was slow (2324 ± 369.6 s, *n* = 4) after a saline injection, reduced by ~ 98% when atipamezole (10 µg/kg) with caffeine (25 mg/kg) were injected (41.75 ± 8, *n* = 4) and reduced by ~ 97% when high-dose atipamezole (100 μg/kg) was injected (63 ± 16.95, *n* = 4). Both atipamezole (10 μg/kg) with caffeine (25 mg/ kg; *p* < 0.0001) and atipamezole (100 μg/kg; *p* = 0.0025) were significantly different than saline but were not different from each other (> 0.99, *n* = 4).

## Discussion

We tested the ability of atipamezole with caffeine to reverse the sedative effects of dexmedetomidine in rats. The goal was to develop a strategy to use low-dose atipamezole to reverse dexmedetomidine sedation and avoid the potential unwanted effects produced by high-dose atipamezole. Atipamezole with either caffeine or forskolin were effective when a bolus of dexmedetomidine was used to sedate rats. Using concentrations of atipamezole 10–20-fold lower than that recommended by the manufacturer, emergence times were 90%-97% shorter than that produced by the saline control. Atipamezole with caffeine was more effective than either atipamezole or caffeine alone. Similarly, atipamezole with forskolin was more effective than forskolin alone.

When used for long-term sedation in mechanically ventilated patients, dexmedetomidine reduces breathing support time and time in ICU. Dexmedetomidine is used as an adjunct to neurosurgery; a list of benefits includes anxiolysis, blood pressure stabilization, analgesia, and sedation without respiratory depression or significant cognitive impairment [1]. Dexmedetomidine is useful in children and is common in procedural sedation, like MRI, with the primary drawback being long recovery times [26]. If found to be effective, atipamezole with caffeine would be useful in these situations.

In a simulated “pediatric MRI” procedure a low dose of atipamezole with caffeine rapidly reversed the dexmedetomidine/ propofol sedation. The rats received 25 μg/kg of dexmedetomidine across the 90 min simulation, which meant that reversal would potentially require 250 μg/kg of atipamezole. For the data shown in Figs. 7 and 6 μg/kg atipamezole was employed, an ~ 42-fold lower dose than recommended. Ongoing metabolism of dexmedetomidine meant that the sedative levels were less than the 25 μg/kg infused. Using an elimination abo-life of ~ 65 min [22], suggests that a significant fraction of the dexmedetomidine was metabolized. Assuming that half the dexmedetomidine was still present, that still is an atipamezole concentration ~ 20-fold lower than the manufacturer’s recommended dose. This result suggests the low dose atipamezole and caffeine is effective in a situation similar to that when dexmedetomidine is used for pediatric MRI sedation.

In 2016 the FDA issued an advisory warning against possible neurotoxic effects of anesthetics and sedatives (www.fda.gov/Drugs/DrugSafety/ucm532356.htm). Exposing the embryos or neonates of different animal species, including non-human primates, to different anesthetics resulted in augmented neuronal apoptosis and developmental/ behavioral abnormalities [7]. Dexmedetomidine is popular in the pediatric population because it is not associated with neurotoxic effects [27]; dexmedetomidine is thought to be neuroprotective [28]. In the elderly, delirium after anesthesia is common and dexmedetomidine may reduce the incidence of these events [9, 10]. We observed no behavioral issues during emergence in our studies.

α_2_ receptors are G-protein coupled receptors linked to the G_i/o_ signaling pathway [4]. Activation of α_2_ receptors results in the inhibition of adenylate cyclase which reduces intracellular cAMP levels [29]. Activation of the G_i/o_ signaling pathway also activates GIRK K^+^ ion channels [30] and inhibits voltage gated Ca^2+^ channels [31]. Activation of the G_i/o_ pathway leads to neuronal inhibition. Drugs that restore cAMP levels, like caffeine, partially reverse G_i/o_ mediated effects. Caffeine elevates intracellular cAMP levels by inhibiting phosphodiesterase [19], thereby preventing the breakdown of cAMP. Caffeine also elevates [Ca^2+^]_i_ and blocks all four adenosine receptors [19]. We previously showed that caffeine at the dose employed in this study accelerated emergence from isoflurane and propofol anesthesia without heart rate and blood pressure changes in rats [32]. Furthermore, caffeine sped emergence from isoflurane anesthesia with minimal hemodynamic effects in healthy human volunteers [33]. Our recent study showed that caffeine reverses light anesthesia but not deep anesthesia in rats [25].

Forskolin activates the enzyme adenylate cyclase thereby elevating [cAMP]_i_ [20, 34]. Unlike caffeine, forskolin does not block adenosine receptors nor produce any of the other changes associated with caffeine. The only point of intersection between the two drugs is that they both elevate intracellular cAMP, suggesting that this mechanistic pathway underlies the reversal of dexmedetomidine sedation observed.

In earlier studies, we saw that agents that produced an elevation in intracellular cAMP levels, like caffeine, theophylline and forskolin, all accelerated emergence from anesthesia [32]. A small amount (0.1 ml) of DMSO was needed to dissolve forskolin. This amount of DMSO, by itself, did not affect emergence from anesthesia or cause adverse effects in rats (Wang et al., 2014). Other stimulants, like methylphenidate and amphetamine, may have similar actions [35]. The strategy used in this study may work for these other stimulants. Strong stimulants by themselves reverse deep dexmedetomidine sedation [36]. A different strategy would require a modest dosage of atipamezole and the stimulant. This strategy may prevent possible unwanted effects due to the use of high doses of long-lasting stimulants.

Atipamezole effectively antagonizes dexmedetomidine [17]. Atipamezole is widely used in veterinary medicine [14], where it reverses dexmedetomidine anesthesia. Atipamezole is not used in the human population [15, 16] as human clinical trials suggested that doses of atipamezole up to 30 mg per subject produced few cardiovascular or subjective side effects, while higher doses (up to 100 mg per subject) produced unwanted symptoms, such as emesis, motor restlessness, and clinically relevant increases in blood pressure [13, 16, 17]. In humans, effective reversal of dexmedetomidine sedation needed atipamezole dosages 40–100-fold higher than that of dexmedetomidine [18]. Atipamezole alone cannot effectively reverse dexmedetomidine sedation in humans since the high dosages needed are associated with unwanted side effects. Using a low-dose of atipamezole with caffeine in humans similar to that shown in the current study should not be associated with unwanted effects since atipamezole levels remain modest at all times.

Each rat used in this study was exposed to dexmedetomidine multiple times. Each exposure produced the same effect. No tolerance to the drug was observed.

Although low-dose atipamezole alone accelerated emergence from anesthesia (Figs. 2 and 3), “emergence times” were slow with considerable variability in emergence times between rats. Some rats emerged from sedation very slowly even in the presence of low-dose atipamezole. Only low-dose atipamezole with caffeine guaranteed rapid and consistent emergence.

Throughout this study, 25 mg/ kg caffeine, was employed as a reversal agent, either by itself or in combination with atipamezole. Undoubtedly, a higher dose of caffeine would have been even more effective. Our goal in this study was to use relatively modest doses of both atipamezole and caffeine so as to avoid any side effects from either drug. We have previously tested the ability of caffeine citrate to accelerate emergence from isoflurane anesthesia in human test subjects [33]. In that study we observed that 15 mg/ kg caffeine citrate in human test subjects was equivalent in terms of effectiveness to 25 mg/ kg caffeine base in rats [33]. This is a safe dose of caffeine for both humans and rats and is less than the 20 mg/ kg caffeine loading dose used in neonates suffering from apnea of prematurity [37].

A recent study showed that dexmedetomidine produced a more profound sedative effect in female than male rats [36]. In our study, the sedative effects produced by dexmedetomidine was not significantly different between male and female rats, although a larger group size may be needed to uncover a difference.

Finally, although rare, patients can get an overdose of dexmedetomidine. The results outlined in this study suggest that the combination of atipamezole with caffeine may be an effective rescue drug for these rare situations.

For dexmedetomidine sedation, prolonged recovery and hemodynamic changes, particularly bradycardia, are important drawbacks [23, 38]. Our results suggest that low dose atipamezole with caffeine was able to rapidly restore heart rate to pre-dexmedetomidine levels. Our results also showed that low-dose atipamezole with caffeine were not associated with a change in blood pressure.

### Limitations of the study

We tested a single dosage of caffeine in this study because caffeine at this dosage did not change vital signs in rats, yet it was able to accelerate emergence from anesthesia (Wang et al. 2014). We used forskolin in only one group of rats as repeatedly use of DMSO may be harmful to the rats. Rats and humans show markedly different physiological responses: drugs that work in rats can fail in human clinical trials. As an example, the drug fialuridine, developed to hepatitis B, worked well in mice and rats and dogs but humans developed liver failure and five died [39]. Even a transient hypotension evoked by atipamezole in rodents is not seen in humans [40]. The results presented in the current manuscript must be considered preliminary until confirmed in the human population.

## Conclusion

In summary, we show in rats that a combination of atipamezole with caffeine or forskolin effectively reversed dexmedetomidine-induced sedation up to dosages of 40 μg/kg. This strategy awaits testing in the human population.

## Data Availability

With the exception of vital sign data, all other data is included in the manuscript itself (see figure legends). Vital sign data will be made available upon request.
